# The Importance of Amino Acid Composition in Natural AMPs: An Evolutional, Structural, and Functional Perspective

**DOI:** 10.3389/fimmu.2012.00221

**Published:** 2012-07-31

**Authors:** Biswajit Mishra, Guangshun Wang

**Affiliations:** ^1^Department of Pathology and Microbiology, University of Nebraska Medical CenterOmaha, NE, USA

Antimicrobial peptides (AMPs) are critical components of natural host defense systems against infectious pathogens (Zasloff, 2002; Boman, 2003; Hancock and Sahl, 2006). They are ubiquitous in nature and have been found in nearly all forms of life, ranging from single-celled bacteria to multicellular organisms such as plants and animals. AMPs are short peptides (5–100 amino acids) with an average net charge of +3 (Wang, 2010). They can display broad or narrow-spectrum antimicrobial activities. The fact that AMPs are effective against multidrug resistance pathogens, including suppression of biofilm formation, deserves our attention (Menousek et al., 2012). In addition to direct bacterial elimination, these peptides have regulatory effects on immune systems. Consequently, AMPs are also referred to as host defense peptides (Hancock and Sahl, 2006). To decode the key elements behind the functional diversity of AMPs, we have been taking time and efforts in constructing a comprehensive database that annotates such information. The first version of the Antimicrobial Peptide Database (APD; http://aps.unmc.edu/AP/main.html) was established in 2003 (Wang and Wang, 2004) and the database has since been further developed (Wang et al., 2009). The APD contained 1973 entries as of May 2012. To facilitate our bioinformatic analysis, we will register a peptide into the APD if it is (1) from natural sources; (2) with minimal inhibitory concentration (MIC) of less than 100 μM or 100 μg/mL; (3) less than 100 amino acid residues; and (4) with a characterized amino acid sequence (Wang, 2010). The APD allows users to extract important parameters (e.g., charge, hydrophobicity, motif, and structure) that determine peptide function. In particular, our database enables the generation of the amino acid composition for a select peptide or a family of AMPs with a common feature. This bioinformatic tool thus uncovers the amino acid use in natural AMPs from different sources, with different functions, or three-dimensional structures. This opinion article highlights the critical roles of the amino acid composition in naturally occurring AMPs in terms of evolutional, structural, and functional significance. Moreover, its application in designing and predicting new AMPs will also be discussed.

## Evolutionary Perspective

**Figure 1:**
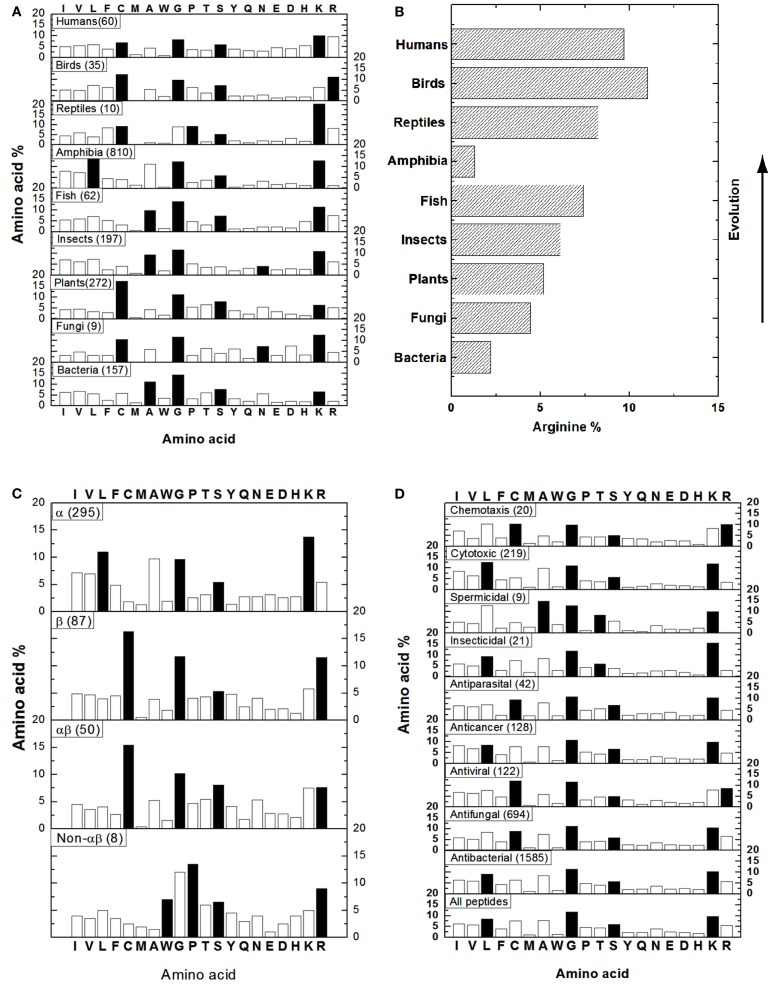
**Amino acid composition analysis of natural AMPs based on source (A,B), structure (C), and activity (D)**. **(B)** Shows the arginine percentages of AMPs from different life domains. In each case, the number of peptides included in the bioinformatic analysis is given in parentheses. The solid bars represent the most abundant amino acids in the hydrophobic (I, V, L, F, C, M, A, W), GP (G and P), polar (T, S, Y, Q, N), and charged (E, D, H, K, R) groups. Data were obtained in May 2012 (total peptides: 1973) from http://aps.unmc.edu/AP/main.php by using the search and statistical analysis functions of the APD (Wang et al., 2009).

To get an idea of AMPs in different kingdoms, we obtained the amino acid composition profiles of these peptides from bacteria, fungi, plants, insects, fish, amphibia, reptiles, birds, and humans (Figure 1A) by performing source search in the APD (Wang et al., 2009). In our database, the 20 standard amino acids are classified into four groups: hydrophobic (I, V, L, F, C, M, A, and W), GP (G and P), polar (T, S, Y, Q, and N), and charged (E, D, H, K, and R; Wang and Wang, 2004). In Figure 1, the dominant amino acids (highest percentages) in the four groups are represented as solid bars. For bacterial AMPs (i.e., bacteriocins), alanines (A) are the most preferred hydrophobic amino acid while residues G, S, and K are the most abundant in the other three groups (Figure 1A). Similarly, A is also a dominating hydrophobic residue in AMPs from insects or fish. In amphibian AMPs, L is the most abundant hydrophobic residue. In contrast, C is the major hydrophobic residue in AMPs from fungi, plants, and birds, probably due to the dominance of disulfide bonded defensin-like molecules. In human or reptile AMPs, C is comparable to other hydrophobic residues (e.g., L), probably reflecting the diversity in peptide sequences. For example, the known human AMPs are defensins, cathelicidins, histatins, and β-amyloid peptides. Like the case of bacteria, G, S, and K are usually the dominant residues in the other three amino acid groups in Figure 1A. Exceptions are as follows. In the case of reptile AMPs, G and P are comparable. For AMPs from fungi and insects, the level of N is higher than or similar to S. Different from other kingdoms, birds select arginines (R) as the main charged amino acid, whereas arginines and lysines (K) are comparable in human AMPs. Based on the above description, it is clear that the dominant hydrophobic amino acids differ in various kingdoms, while residues G, S, and K are generally preferred amino acids in natural AMPs from nearly all the kingdoms (Figure 1A). The variations in the dominant amino acids in the hydrophobic group are an important observation and could suggest the preference of specific types of AMPs in certain kingdoms. In addition, one of the most important aspects is the observation made by Torrent et al. (2011) also on the basis of the APD (Wang et al., 2009). They found that higher organisms tend to incorporate R more frequently than K except amphibians (Figure 1B). The authors attributed this phenomenon to the possible emergence of the adaptive immune systems and the arginine-rich AMPs may well play an important role in modulating the immune system and in linking the innate and adaptive immune systems.

## Structural Insight

It is now clear that AMPs can adopt a variety of fascinating scaffolds, ranging from linear to circular. However, there are only four types of structures based on secondary structures: α, β, αβ, and non-αβ (Wang, 2010). The α family consists of AMPs with α-helical structures, while the β family comprises AMPs with a β-sheet structure. Another two families can be understood accordingly: αβ = α + β and non-αβ = no α and no β structure. Representative structures for these four families can be viewed at the face page of the APD website above. The APD has also annotated those AMPs with determined 3D structures, which form the basis for our amino acid analysis. The peptides belonging to the α family are widely distributed in bacteria and animals, while most of the plant AMPs, such as cyclotides and defensins, belong to the β family. The αβ members occur in all kingdoms, including bacteria, plants and animals, but the non-αβ AMPs are much less frequent and are only confined to the animal kingdom at present. Depending on the structural family, the dominant amino acid in each amino acid group differs (Figure 1C). The α helical AMPs prefer L as the major hydrophobic amino acid while K is selected as the charged amino acid. On contrary, the β stranded AMPs are dominated by C that determines the polypeptide fold. Meanwhile, it prefers R instead of K as the charged amino acid. Likewise, the αβ family has a high content of C as the hydrophobic component required for peptide folding. However, it possesses equal amounts of R and K. Finally, the non-αβ family is generally composed of AMPs that are rich in particular amino acids such as tryptophan (W), proline (P), and R. The G and S are the other two preferred amino acids in all the families (Figure 1C). It is evident that amino acid composition is related to 3D structure of natural AMPs (Wang et al., 2009).

## Functional Implications

Natural AMPs with either narrow or broad-spectrum activity have been reported. Moreover, there is overlap in the activity spectrum of some AMPs (Zasloff, 2002; Hancock and Sahl, 2006). Such an activity spectrum for each AMP has been annotated in the APD. This includes antibacterial, antifungal, antiviral, antiparasital, insecticidal, spermicidal, anticancer, cytotoxic (e.g., hemolytic), and chemotactic activity (Wang and Wang, 2004; Wang et al., 2009). Figure 1D shows the distribution of amino acids based on peptide activity. Except for spermicidal and insecticidal peptides, where T is preferred in the polar group, amino acids G and S are the two representative residues in the GP and polar amino acid groups in all the cases. For hydrophobic residues, L is the dominant amino acid for AMPs with cytotoxic, insecticidal, anticancer, or antibacterial activity. There is a subtle difference between AMPs active against Gram-positive and Gram-negative strains only. AMPs active against Gram-positive bacteria have similar contents of C and L, whereas those against only Gram-negative AMPs have higher L and lower C contents (not shown). Residue C is dominant in the hydrophobic group for AMPs with chemotactic, antiparasital, antiviral, or antifungal activity, suggesting the existence of a significant number of disulfide bonded molecules. In the case of spermicidal AMPs, residue A is the major hydrophobic amino acid. Finally, while lysines are usually the positively charged residue, arginines are clearly important in AMPs with chemotactic and antiviral activities. Therefore, the amino acid composition plays a role in determining peptide activity as well. For example, anticancer peptides are rich in L, G, S, and K, whereas chemotactic peptides have high C, G, T, and R contents.

## Applications of Abundant Amino Acids in Peptide Design

As recently summarized by Wang (2010), there are various methods in designing new AMPs, ranging from template optimization, motif hybridization, sequence shuffling/library screening, to rationale design. It has been recognized that parameters such as charge and hydrophobicity play a tremendous role in determining AMP activity (Zasloff, 2002; Hancock and Sahl, 2006; Wang, 2010). Our opinion is that the abundant residues identified in amino acid composition profiles of AMPs (Figure 1) can be used to design a specific peptide with the *desired* activity. Indeed, we succeeded in designing a 19-residue peptide using only residues G, L, and K. GLK-19 is active against *E. coli* but not *S. aureus* (Wang et al., 2009) or HIV-1. Since antiviral AMPs prefer arginines (Figure 1D), we obtained an anti-HIV peptide GLR-19 after the conversion of lysines in GLK-19 to arginines (Wang et al., 2010). In addition, because C is the dominant hydrophobic residue in antiviral peptides, we further improved anti-HIV activity of the peptide after introduction of a pair of cysteines to GLR-19 between residues 4 and 16 (Wang et al., 2011). Therefore, we succeeded in modulating peptide activity by varying the amino acid composition. We also propose that the prediction interface of the APD can be improved based on the abundant amino acids identified herein.

## A Summary of Opinions

The construction of the APD made it possible for us to extract the amino acid composition information in natural AMPs for the first time. Further classification of AMPs and the update of our database made the extracted parameters more informative (Wang et al., 2009). We propose that the amino acid composition plays an important role in terms of evolution, structure, and function of natural AMPs. The overall picture for natural AMPs is shaped through evolution. For example, the preference of arginines in the AMPs in higher organisms (Figure 1B) is proposed to be significant in the emergence of adaptive immune systems (Torrent et al., 2011) and probably also confers the regulatory and integrative role to natural AMPs in host defense. We demonstrated that arginines are more effective in targeting MRSA or HIV-1 (Wang et al., 2010, 2012). The amino acid composition appears to directly determine the various structural scaffolds of natural AMPs (Figure 1C). In the case of amphibians, the dominance of G, L, A, and K determines a helical conformation, leading to a natural recombinant library of peptides (up to ∼100 in each frog) achieved by presenting varying other amino acids on the same helical structure backbone (Wang et al., 2009). Likewise, plant cyclotides are rich in C, G, T/S, and K that determines a universal β-sheet containing scaffold (Wang, 2010). Again, nature has created a natural recombinant library of cyclotides by introducing other amino acids to various loop regions. The same strategy is now utilized to generate new cyclotides with a desired biological function via segment grafting (Craik et al., 2012). Amino acid compositions may determine the mechanisms of action of natural AMPs. As we noticed previously, plant cyclotides and bacterial lantibiotics have different structural folds but similar amino acid composition profiles (Wang, 2010). Interestingly, certain lantibiotics can bind phosphatidylethanolamines (PE; Zhao, 2011; Ökesli et al., 2011), so can cyclotides (Henriques et al., 2011). There is now converged view regarding the mechanism of action of proline-rich AMPs. They can cross bacterial membranes and associate with heat-shock proteins (Scocchi et al., 2011). The abundant amino acids elucidated from the APD (Figure 1) are helpful for both prediction and design of new AMPs (Wang, 2010). In our opinion, database-guided design is preferred over library screening due to its cost effectiveness by synthesizing only few peptides. Future database annotations and expansion are anticipated to further improve the accuracy of the amino acid composition profiles, thereby opening the door to other potential applications.
